# Genomic Prediction Enhanced Sparse Testing for Multi-environment Trials

**DOI:** 10.1534/g3.120.401349

**Published:** 2020-06-11

**Authors:** Diego Jarquin, Reka Howard, Jose Crossa, Yoseph Beyene, Manje Gowda, Johannes W. R. Martini, Giovanny Covarrubias Pazaran, Juan Burgueño, Angela Pacheco, Martin Grondona, Valentin Wimmer, Boddupalli M. Prasanna

**Affiliations:** * University of Nebraska – Lincoln, Lincoln NE, 68583,; ^†^ International Maize and Wheat Improvement Center (CIMMYT), Km. 45, Carretera México-Veracruz, El Batán, Texcoco, Edo. de México, CP 56130, México; CIMMYT, P. O. Box 1041, Village Market, 00621, Nairobi, Kenya,; ^‡^ Colegio de Postgraduados, Montecillos, Edo. de Mexico, Mexico,; ^§^ Advanta Seed, College Station, Texas A&M, Texas, and; ** KWS SAAT SE & Co. KGaA, Grimsehlstr. 31, 37574 Einbeck, Germany

**Keywords:** genomic-enabled prediction accuracy, sparse testing methods, allocation of non-overlapping/overlapping genotypes in environments, random cross-validations, maize multi-environment trials, genotype-by-environment interaction GE, GenPred, Shared data resources

## Abstract

“Sparse testing” refers to reduced multi-environment breeding trials in which not all genotypes of interest are grown in each environment. Using genomic-enabled prediction and a model embracing genotype × environment interaction (GE), the non-observed genotype-in-environment combinations can be predicted. Consequently, the overall costs can be reduced and the testing capacities can be increased. The accuracy of predicting the unobserved data depends on different factors including (1) how many genotypes overlap between environments, (2) in how many environments each genotype is grown, and (3) which prediction method is used. In this research, we studied the predictive ability obtained when using a fixed number of plots and different sparse testing designs. The considered designs included the extreme cases of (1) no overlap of genotypes between environments, and (2) complete overlap of the genotypes between environments. In the latter case, the prediction set fully consists of genotypes that have not been tested at all. Moreover, we gradually go from one extreme to the other considering (3) intermediates between the two previous cases with varying numbers of different or non-overlapping (NO)/overlapping (O) genotypes. The empirical study is built upon two different maize hybrid data sets consisting of different genotypes crossed to two different testers (T1 and T2) and each data set was analyzed separately. For each set, phenotypic records on yield from three different environments are available. Three different prediction models were implemented, two main effects models (**M1** and **M2**), and a model (**M3)** including GE. The results showed that the genome-based model including GE (**M3**) captured more phenotypic variation than the models that did not include this component. Also, **M3** provided higher prediction accuracy than models **M1** and **M2** for the different allocation scenarios. Reducing the size of the calibration sets decreased the prediction accuracy under all allocation designs with **M3** being the less affected model; however, using the genome-enabled models (*i.e.*, **M2** and **M3**) the predictive ability is recovered when more genotypes are tested across environments. Our results indicate that a substantial part of the testing resources can be saved when using genome-based models including GE for optimizing sparse testing designs.

Multi-environmental trials (METs) that allow assessing the performance of different candidate genotypes under varying environmental conditions are essential components of breeding schemes. Estimating genotype × environment (GE) interaction is important to identify stable genotypes or genotypes with specific adaptation. The environments can be given by managed stress trials, but can also simply be defined by different locations without clear distinction between the types of conditions. Ideally, all genotypes under consideration should be observed at each location to investigate the GE interactions of the germplasm. This approach requires extensive field-testing (Smith *et al.* 2015a; Smith *et al.* 2015b).

In the last two decades, genome-based prediction of genetic values (GP, Meuwissen *et al.* 2001) has revolutionized plant and animal breeding (Hayes *et al.* 2009; Jannink *et al.* 2010; Crossa *et al.* 2010, 2011; Crossa *et al.* 2017). GP uses dense molecular markers from the entire genome to derive a genomic relationship matrix which can be used to predict the performance of lines of known genotype but unknown phenotype. GP has been used to increase selection accuracy by using predictions as additional (multi-year or multi-location) phenotypes (Jarquín *et al.* 2014a), to reduce the cycle length by skipping certain stages (Crossa *et al.* 2017) or to reduce the experimental effort by using only testing subsets of the considered genotypes, thus increasing the evaluation capacity and, potentially, the selection intensity.

The latter is of particular interest in the context of METs. Here, predictions can reduce the experimental effort by using “sparse testing” methods in which only a subset of the genotypes that we wish to evaluate is tested at each location. The missing (unobserved) genotype-in-environment combinations can be predicted from the measured data. Sparse testing reduces the costs at a fixed evaluation capacity, or increases the overall evaluation capacity at fixed costs, thus leading to an increase in selection intensity or an increase in accuracy by better coverage of the target population of environments (TPE) and, potentially, increasing the selection gains. Here - as often occurs when dealing with the breeder’s equation - we are facing a trade-off between two components. The prediction may be less accurate than a measured phenotype, but an increase in selection intensity may compensate for the loss of accuracy by far and ultimately lead to an overall increase in selection gain (Fehr 1987). Therefore, a crucial question is which design (that is, how the genotypes should be partitioned across environments) gives the best relationship between accuracy and evaluation capacity.

The predictive ability of methods is usually evaluated using some form of cross-validation (CV) that splits the observed data set into a calibration (training) set and a prediction (testing) set, predicting the phenotypic performance of the genotypes in the prediction set by using the phenotypes of the calibration set. In order to evaluate the model’s performance, the predicted values of the genotypes in the prediction set are compared to their observed phenotypes. In the context of structured data consisting of year cohorts with phenotypes obtained in different environments, different types of CVs mimicking potential applications are conceivable. For instance, Burgueño *et al.* (2012) studied the prediction accuracy when predicting the performance of genotypes that had never been evaluated (named cross-validation 1, CV1). For CV1, the phenotypic records of other genotypes grown in the relevant environments are used as a calibration set. An alternative is cross-validation 2 (CV2) in which the performance of some genotypes in specific environments is predicted by a calibration set which includes records of the same genotype in other environments. CV2 represents the problem of predicting a certain portion of tested genotypes in a certain portion of tested environments (incomplete field trials).

These CV schemes (CV1, CV2) represent sparse testing designs with different levels of overlapping genotypes. Sparse testing approaches are particularly useful in early generation testing when a large number of genotypes is available (Butler *et al.* 2014; Oakey *et al.* 2016). Here, a crucial question is how to design a multi-environmental trial system that will optimize the trade-off between the selection intensity (number of genotypes tested) and the accuracy of the predicted values. Due to the generally limited resources, this leads to a resource allocation problem for maximizing genetic gain at fixed costs.

The aim of this study is to investigate how a set of genotypes can be arranged across different environments, given a total number of plots. We varied the number of overlapping genotypes with the objective of improving the predictive ability of untested genotype-by-environment combinations. We studied the two extreme cases of (1) non-overlapping genotypes between environments (NO) with each line being observed exactly once across environments and (2) the same set of genotypes being tested in all environments (“all overlapping”). Since the overall number of plots has been fixed, all other genotypes to be predicted have never been observed in any location for scenario (2). Between these two extreme cases, we (3) varied the number of non-overlapping (NO)/overlapping (O) lines. We used two data sets of maize genotypes crossed with two testers: T1 and T2. The two data sets (DST1 and DST2) created using testers T1 and T2 are not-overlapping in terms of the lines used for the crosses. In all cases we fitted three different prediction models as follows: (M1) including only the environment and genotype main effects (no molecular marker information nor any interaction was included); (M2) environmental, genotype and genomic main effects; and (M3) environmental, genotype, genomic main effects and GE interaction.

## Materials And Methods

### Maize experimental multi-environment data sets

For this study, we used two maize data sets from CIMMYT’s maize breeding program in eastern Africa: DST1 comprised 843 unique CIMMYT maize genotypes where 843 unique genotypes were crossed with tester T1, while DST2 had 453 CIMMYT maize genotypes where 453 unique genotypes were crossed with tester T2. For both data sets, genotypic data from 73,219 SNP markers were available. After applying conventional quality control on the molecular markers (SNPs with more than 50% missing values and with a minor allele frequency lower than 3% were discarded), the number of SNP markers that remained for analysis were 68,169 and 62,882 for DST1 and DST2, respectively. The genotypes crossed with testers T1 and T2 were different and the data sets have therefore been considered separately.

Data sets DST1 and DST2 consist of hybrids created by the crosses between the unique genotypes and the two testers T1 and T2. The maize hybrids were evaluated in three environments in Kenya, of which two represent optimal conditions and one drought stress. The phenotypic correlations for DST1 were 0.08 and 0.07 between the records of the drought environment and the two optimal locations, and 0.12 between the records from the two optimal locations. The corresponding values for DST2 were higher, with 0.37 and 0.13 for the correlation between the drought environment and the two optimal sites, and 0.30 between the two optimal locations.

### Allocation designs for sparse testing

#### Sampling non-overlapping/overlapping methods for assessing sparse testing:

Suppose we are interested in DST1, where there are a total of 843 maize genotypes that need to be evaluated in three different environments. However, due to budget limitations, the number of plots that can be tested in the field is limited because we cannot test 2,529 (843 × 3) plots representing all the genotypes in all three environments. Then we need to decide whether to test one set of lines across all environments (overlapping), multiple sets of lines within environments (non-overlapping), or a mix between overlapping and non-overlapping lines. We can differentiate between designs by their fraction of numbers of non-overlapping (NO), and overlapping lines (O).

Let us assume that initially we are granted resources for phenotyping only 843 genotypes (1/3 of the total genotype/environment combinations). Further, let us assume that the phenotyping costs are the same in all environments; then the allocation problem is simplified and reduced to deciding how many genotypes will be observed in the different environments.

We denoted the set of genotype-in-environment combinations that are observed in the field as the calibration set. For these genotype-in-environment combinations we have marker and corresponding phenotypic information. We use this information to calibrate the prediction model for predicting the remaining set of untested genotypes-in-environment combinations. The genotype-in-environment combinations for which we obtain the predictions is the prediction set.

**Figure 1:**
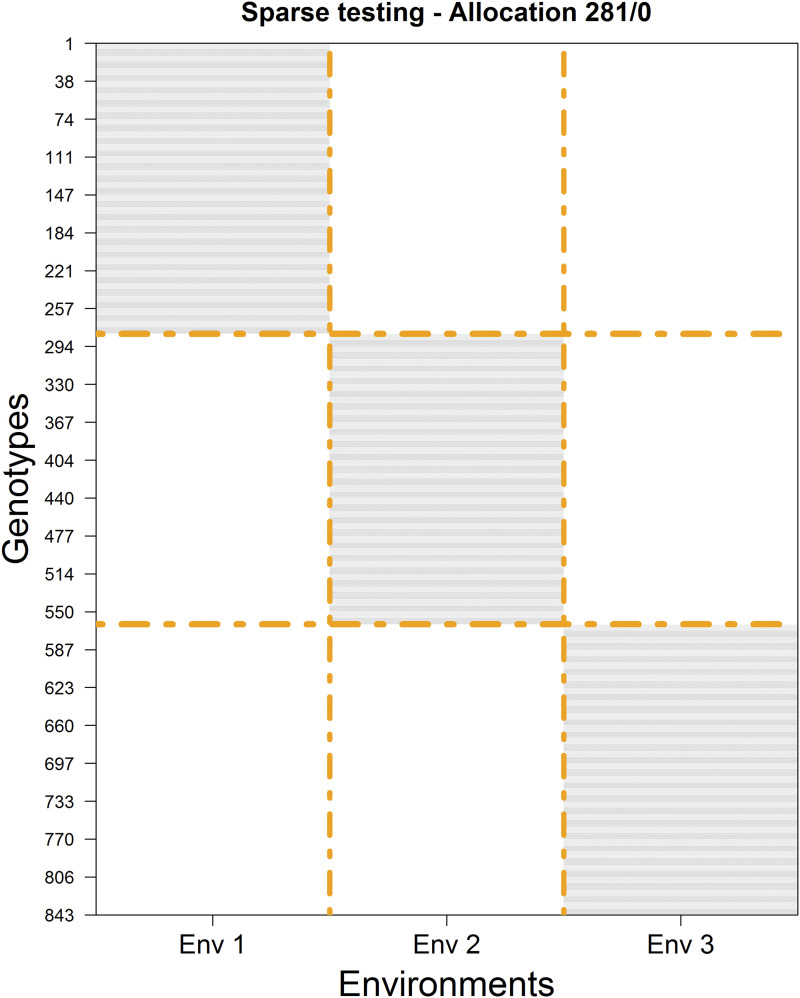
Sparse design for allocating 843 plots to be tested in three environments for 843 unique genotypes with 281 non-overlapping/0 overlapping. Horizontal gray lines indicate the genotype-by-environment combinations that were tested in each environment. The rows correspond to the genotypes (from 1 to 843) while columns represent the environments (from Env 1 to Env 3).

**Figure 2:**
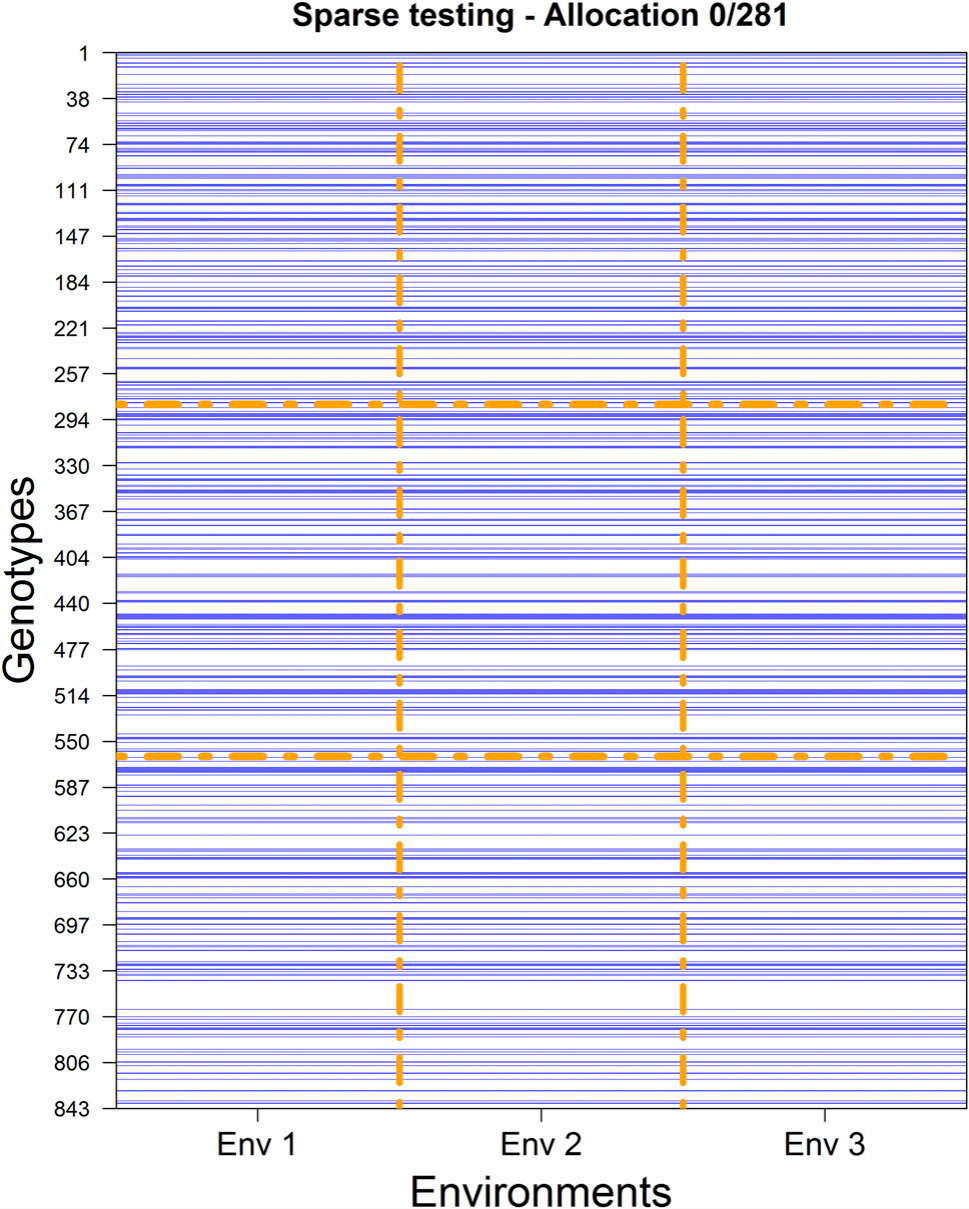
Experimental design for allocating 843 plots to be tested in three environments for 281 unique genotypes. The blue lines correspond to genotypes tested across the three environments (columns).

**Figure 3:**
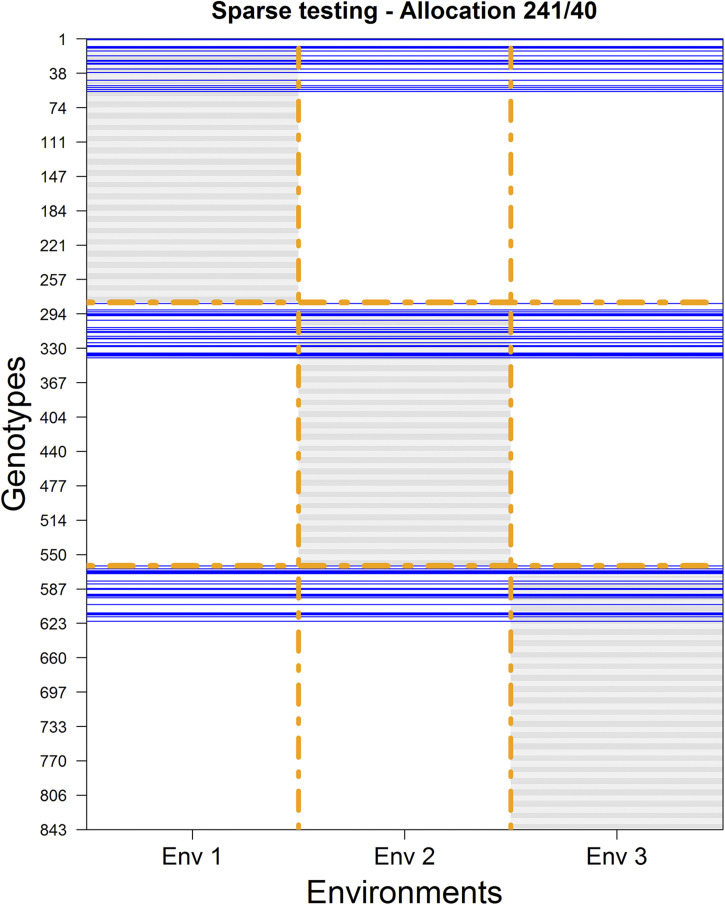
Experimental design for allocating 843 plots to be tested in three environments for 843-80 = 763 unique genotypes. Horizontal gray lines indicate that these genotypes were tested in the corresponding environments (columns). Blue lines correspond to genotypes (40) tested across environments.

The different allocation designs depend on the number of NO/O maize genotypes in each environment. The overlapping genotypes can be considered as a bridge for connecting environments. In order to describe the different designs, we show and explain examples in Figures 1-3 and Table 1. Other scenarios may appear when there is a restriction with respect to the number of genotypes to be tested in each environment.

**Table 1:** Testing composition combinations between non-overlapping and overlapping sets of lines for different sample sizes (SS) to be tested within environments for the case of Maize Tester 1 (843 genotypes, partitioned into 281 non-overlapping set, tested in different environments)

SS per Env	Testing set composition within environment non overlaping/overlaping														
281	281/0	271/10	261/20	251/30	241/40	231/50	221/60	211/70	201/80	191/90	181/100	171/110	161/120	151/130	
271		271/0	261/10	251/20	241/30	231/40	221/50	211/60	201/70	191/80	181/90	171/100	161/110	151/120	
261			261/0	251/10	241/20	231/30	221/40	211/50	201/60	191/70	181/80	171/90	161/100	151/110	
251				251/0	241/10	231/20	221/30	211/40	201/50	191/60	181/70	171/80	161/90	151/100	
241					241/0	231/10	221/20	211/30	201/40	191/50	181/60	171/70	161/80	151/90	
231						231/0	221/10	211/20	201/30	191/40	181/50	171/60	161/70	151/80	
221							221/0	211/10	201/20	191/30	181/40	171/50	161/60	151/70	
211								211/0	201/10	191/20	181/30	171/40	161/50	151/60	
201									201/0	191/10	181/20	171/30	161/40	151/50	
191										191/0	181/10	171/20	161/30	151/40	
181											181/0	171/10	161/20	151/30	
171												171/0	161/10	151/20	
161													161/0	151/10	
151														151/0	
Cont. by column															
281	141/140	131/150	121/160	111/170	101/180	91/190	81/200	71/210	61/220	51/230	41/240	31/250	21/260	11/270	1/280
271	141/130	131/140	121/150	111/160	101/170	91/180	81/190	71/200	61/210	51/220	41/230	31/240	21/250	11/260	1/270
261	141/120	131/130	121/140	111/150	101/160	91/170	81/180	71/190	61/200	51/210	41/220	31/230	21/240	11/250	1/260
251	141/110	131/120	121/130	111/140	101/150	91/160	81/170	71/180	61/190	51/200	41/210	31/220	21/230	11/240	1/250
241	141/100	131/110	121/120	111/130	101/140	91/150	81/160	71/170	61/180	51/190	41/200	31/210	21/220	11/230	1/240
231	141/90	131/100	121/110	111/120	101/130	91/140	81/150	71/160	61/170	51/180	41/190	31/200	21/210	11/220	1/230
221	141/80	131/90	121/100	111/110	101/120	91/130	81/140	71/150	61/160	51/170	41/180	31/190	21/200	11/210	1/220
211	141/70	131/80	121/90	111/100	101/110	91/120	81/130	71/140	61/150	51/160	41/170	31/180	21/190	11/200	1/210
201	141/60	131/70	121/80	111/90	101/100	91/110	81/120	71/130	61/140	51/150	41/160	31/170	21/180	11/190	1/200
Cont. by row															
191	141/50	131/60	121/70	111/80	101/90	91/100	81/110	71/120	61/130	51/140	41/150	31/160	21/170	11/180	1/190
181	141/40	131/50	121/60	111/70	101/80	91/90	81/100	71/110	61/120	51/130	41/140	31/150	21/160	11/170	1/180
171	141/30	131/40	121/50	111/60	101/70	91/80	81/90	71/100	61/110	51/120	41/130	31/140	21/150	11/160	1/170
161	141/20	131/30	121/40	111/50	101/60	91/70	81/80	71/90	61/100	51/110	41/120	31/130	21/140	11/150	1/160
151	141/10	131/20	121/30	111/40	101/50	91/60	81/70	71/80	61/90	51/100	41/110	31/120	21/130	11/140	1/150

#### Allocating 281 non-overlapping/0 overlapping genotypes:

First, for selecting the calibration set, suppose we decide to plant the same number of genotypes per environment (281 = 843 / 3). The next issue to consider is how to select and assign these genotypes to the environments. For example, should we assign non-overlapping/overlapping genotypes across the environments? If so, how many? The simplest design would include 3 non-overlapping sets of genotypes leading to a calibration set of 281 genotypes in each of the three environments (Figure 1). With this allocation design, we ensure that each genotype will be tested (observed) in exactly one environment. Hence, for each environment, the prediction set would be composed of the remaining 562 (2 × 281) genotype-in-environment combinations that were not observed.

#### Allocation design - 0 non-overlapping/281 overlapping genotypes:

The opposite extreme case of 281/0 is the case of 0/281. Here, a common set of 281 genotypes is tested across environments (see Figure 2). The prediction set consists of all genotype-in-environment combinations of those genotypes not tested at all.

#### Allocating 241 non-overlapping/40 overlapping genotypes and other designs:

Another scheme may consider sets of common genotypes across environments to allow connectivity across environments. In this design, instead of having all genotypes tested in one environment, we are going to observe 40 genotypes in all of the environments. Figure 3 shows this scheme where a common set of genotypes (40) is observed across environments (see blue lines). This leads to a situation in which 40 genotypes are observed in all three environments, 723 (=3 × 241) genotypes are observed in only one environment and 80 genotypes are not observed at all. It means that 241 unique genotypes are observed in environment 1, another set of 241 unique genotypes are observed in environment 2, and a third set of 241 unique genotypes are observed in environment 3. The total number of plots to observe is 3 × 40 (common in the three environments) + 3 × 241 (different in the three environments) = 843. Therefore, the calibration set consists of these 843 genotype-in-environment combinations, while across environments, the prediction set consists of the remaining 843 × 2 combinations (shown in Figure 3). Table 1 provides a listing of the combinations considered for DST1 for different sample sizes and fixed number of plots for prediction sets (562 = 2 × 281). For each one of the rows in Table 1, 25 different initial random partitions (repetitions) were performed for the cases 281/0, 271/0, …,141/0; then the NO/O designs were gradually varied by sets of 10 genotypes.

## Statistical Models

### Phenotypic adjustment

Best Linear Unbiased Estimators (BLUEs) of the genotypes were computed using ASReml for R for mixed model analysis (Gilmour *et al.* 2009) of grain yield in each environment. The model used to calculate these BLUEs for each environment was

$$y_{jkl}=\mu +L_{j}+r_{k}+d_{l(r)}+e_{jkl},$$

where $y_{jrm}$ represents the phenotypic trait analyzed (grain yield), $L_{j}$ is the fixed effect of the *j*^th^ genotype/line, $r_{k}$ is the random effect of the *k*^th^ replicate that is independently, identically (*iid*), and normally distributed such that $r=\{r_{k}\}∼$ *N*(0,$\;I\sigma_{r}^{2}$) (where ***I*** is the identity matrix and $\sigma_{r}^{2}$ is the variance among replicates), $d_{l(r)}$ denotes the effect of the *l*^th^ incomplete block within the *r*^th^ replicate assumed to be *iid* and normally distributed such that $d=\{d_{l(r)}\}∼$ *N*(0,$\;I\sigma_{l(r)}^{2}$) with $\sigma_{l(r)}^{2}$ being the variance of the incomplete block within the replicate, $e_{jkl}$ is the random error assumed to be *iid* and also normally distributed such that $e=\{e_{jkl}\}∼$*N*(0,$\;I\sigma_{e}^{2}$), where$\;\sigma_{e}^{2}$ denotes the error variance.

To implement the GP analysis, we used the reaction norm model (Jarquín *et al.* 2014b), which is an extension of the random effect Genomic Best Linear Unbiased Predictor (GBLUP) model where the main effect of genotypes, the main effect of environments, the main effect of markers, and their interactions are modeled using random covariance structures that are functions of the genomic and environmental covariates. Brief descriptions of the prediction models are given below.

### Prediction models

For this study we considered three prediction models in which the first two models only consider main effects, while the last one also considers the interaction between marker genotypes and environments. All models assumed that the components were random effects. For all the models, we treated grain yield as the response. We used these prediction models to evaluate the different sparse testing scenarios, and the prediction accuracy (defined as the Pearson correlation coefficient) was used to compare the models’ performance.

#### Model 1 – Environment and genotype main effects (M1: E+L):

Consider that $y_{ij}$ represents the phenotypic value of the *j*^th^ genotype/line in the *i*^th^ environment and can be explained as the sum of an overall mean (μ), a random effect of the *i*^th^ environment ($E_{i\;}$), a random effect of the *j*^th^ genotype/line ($L_{j}$) plus a random error term ($e_{ij}$) capturing the variability not explained by the previous model terms. Also consider that all random effects follow independent and identically (*iid*) normal distributions such that $E_{i}\overset{iid}{∼}N(0,\sigma_{E}^{2})$, $L_{j}\overset{iid}{∼}N\left(0,\sigma_{L}^{2}\right),$ and $e_{ij}\overset{iid}{∼}N\left(0,\sigma_{e}^{2}\right).$ Thus, the model derived from the previous assumptions can be written as follows:

$$y_{ij}=\mu +E_{i}+L_{j}+e_{ij}.$$ (2)

#### Model 2 – Environment, genotype, and genomic main effects (M2: E+L+G):

This model is an extension of M1; it considers the inclusion of the genomic information (marker SNPs) of the genotypes via the score ${\text{g}}_{j}$, which represents the genetic value of the *j^th^* genotype/line. This model component can be defined by the regression on *p* marker covariates ${\text{g}}_{j}=\overset{p}{\underset{m=1}{\sum }}x_{jm}b_{m}$, where $x_{jm}$ is the genotype of the *j^th^* genotype/line at the *m^th^* marker, and $b_{m}$ is the effect of the *m^th^* marker. Assuming that $b_{m}\overset{iid}{∼}N(0,\sigma_{b}^{2})$ (*m=*1,…,*p)*, with $\sigma_{b}^{2}$ being the variance of the marker effects, the vector $g=\left({\text{g}}_{1},\ldots ,{\text{g}}_{J}\right)\text{'}$ follows a multivariate normal density with zero mean and variance-covariance matrix $Cov\left(g\right)=G\sigma_{\text{g}}^{2}$. The term $G\propto \frac{XX^{’}}{p}$ is the genomic relationship matrix and it corresponds to the matrix computed using method 1, as proposed by VanRaden (2008). The entries of the G matrix describe the genomic similarities between pairs of genotypes, **X** is the standardized (by columns) matrix of molecular markers and $\sigma_{\text{g}}^{2}=p\sigma_{b}^{2}$ is the genomic variance. The resulting model is

$$y_{ij}=\mu +E_{i}+L_{j}+{\text{g}}_{j}+e_{ij}$$ (3)

with $g=\left\{{\text{g}}_{j}\right\}$, the vector of genomic effects, following a normal density $N(0,G\sigma_{\text{g}}^{2})\;$ and the other terms are as previously defined. This model allows the borrowing of information between genotypes via the matrix of genomic similarities, which makes it possible to predict genotype performance of untested genotypes across environments. This is useful for all the different non-overlapping/overlapping sets, but in particular in those cases where the number of common genotypes across environments increases (*i.e.*, NO/all O, as well as the intermediate cases). It should be pointed out that the main motivation for keeping both effects, $L_{j}$ and ${\text{g}}_{j},\;$in model **M**2 is to account for, as much and as best as possible, imperfect marker information.

#### Model 3 – Environment, genotype, genomic, and genomic × environment interaction effects (M3: E+L+G+GE):

By adding the interaction between markers and environments ($\text{g}E_{ij}$) to **M2**, the model becomes

$$y_{ij}=\mu +E_{i}+L_{j}+{\text{g}}_{j}+\text{g}E_{ij}+e_{ij},$$ (4)

where the $\text{g}E_{ij}$ term corresponds to the interaction between the genetic value of the *j*^th^ genotype in the *i*^th^ environment. This interaction term is assumed to follow a multivariate normal distribution such that $gE∼N\left(0,\left(Z_{g}GZ_{g}^{'}\right){}^{\circ}\left(Z_{E}Z_{E}^{'}\right)\sigma_{gE}^{2}\right)$ (Jarquín *et al.* 2014b). Matrices $Z_{g}$ and $Z_{E}\;$are the incidence matrices for connecting phenotypes with genotypes and the environments, respectively, $\sigma_{\text{g}E}^{2}$ is the variance component of $\text{g}E_{ij}$, and ‘°’ represents the Hadamard product (element-by-element product) between the two matrices.

### Prediction assessment by cross-validation considering non-overlapping/overlapping genotypes in environments

In order to assess the levels of predictive ability that can be accomplished using different strategies (design-model combinations), a cross-validation study is conducted. The phenotypic information for all the genotype-in-environments combinations is known and a portion of these are masked as missing values according to the different designs.

Cross-validation scheme CV2 evaluates the prediction accuracy of models when some genotypes have been evaluated in some environments but not in others. Here, the information from related genotypes (genomic similarities) and correlated environments (replicates) is included, and thus the predictive ability benefits from borrowing information from genotypes within an environment, from genotypes across environments, and from correlated environments (Burgueño *et al.* 2012). On the other hand, CV1 corresponds to the case where certain percentages of genotypes were never tested and are predicted by other genotypes that were field evaluated. Our NO/O allocation schemes studied the gradual changes from the CV2 scheme to the CV1 scheme via the random cross-validations by making small changes in the number of non-overlapped and overlapped genotypes in environments.

For example, the described procedure for allocating the testing set in environments depicted in Figure 1 (where non-overlapped sets were considered) is a particular case of the CV2 scheme where the genotypes were observed in only one environment (zero NO/all O); it corresponds to the diagonal of the matrix depicted in Table 1. The procedure in Figure 3 is another particular case of the CV2 scheme, where around 14% (41/281 × 100) of the genotypes was observed across all environments. In this study, we considered a comprehensive and exhaustive overlapping set of the genotypes across environments varying between 3.6% (almost all NO) and 99.6% (almost all O).

The random cross-validation scheme, CV1, considers the problem of predicting ‘newly’ developed genotypes/lines that have not yet been observed in any field. Here, the prediction accuracy relies mostly on the genomic relationships between genotypes in the testing and prediction sets. Figure 2 provides an example of this scheme where a common set of 280 genotypes was observed across environments. Although in this study we did not target this CV1 scheme, results derived from the last column in Table 1 could lead to similar outcomes because the levels of non-overlapping genotypes are reduced or close to being null (less than 1%). Thus, Table 1 shows extensions of CV1 and CV2 applied to cases with different NO/O allocation.

The prediction accuracy was measured on a trial basis as the Pearson correlation coefficient between the observed (BLUEs) and predicted values within environments. For data sets DST1 and DST2, the sample sizes of the genotypes in the prediction set within environments were different: 562 (843-281) (in DST1) and 302 (453-151) (in DST2).

### Data availability

The phenotype and genotype data from the genotypes crossed with the testers (data sets DST1 and DST2), as well as other complete tables with the genomic-enabled prediction accuracy in each of the three environment for DST1 and DST2, can be downloaded from the following link http://hdl.handle.net/11529/10548369

### Software

The genomic prediction analyses were computed using R and the models were fitted using the BGLR package (Pérez and de los Campos 2014).

## Results

**Figure 4:**
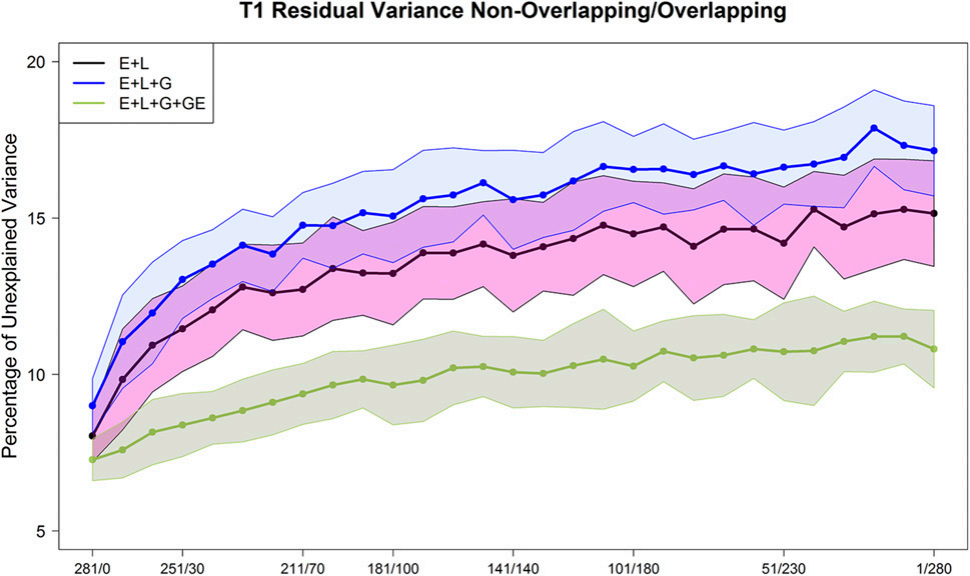
Maize data set DST1. Percent of the unexplained variance (residual variance) for the 3 models M1 (E+L), M2 (E+L+G), and M3 (E+L+G+GE) for different composition of the non-overlapped/overlapped allocation designs. Shaded areas represent the interval of the mean plus minus one standard deviation.

**Figure 5:**
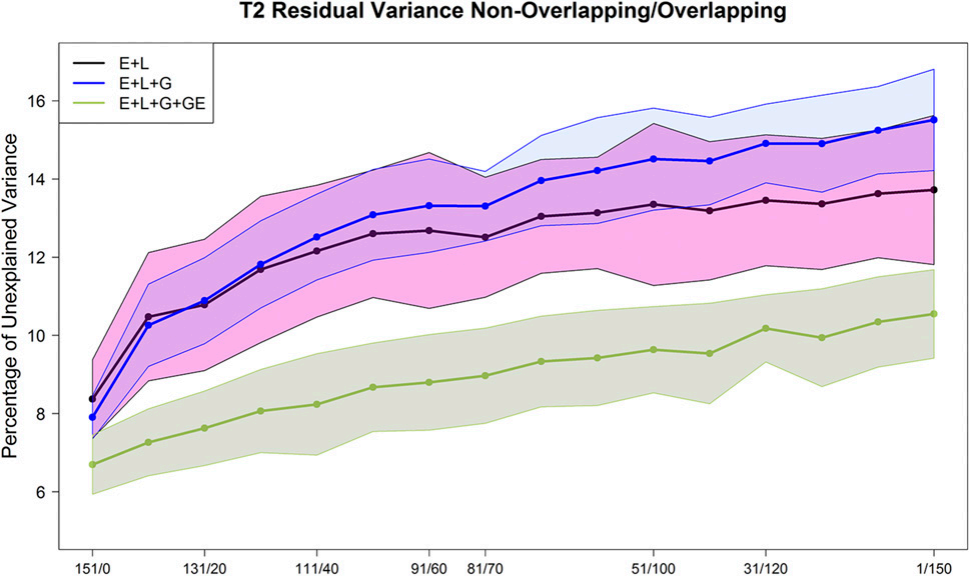
Maize data set DST2. Percent of the unexplained variance (residual variance) for the 3 models M1 (E+L), M2 (E+L+G), and M3 (E+L+G+GE) for different composition of the non-overlapped/overlapped allocation designs. Shaded areas represent the interval of the mean plus minus one standard deviation.

**Figure 6:**
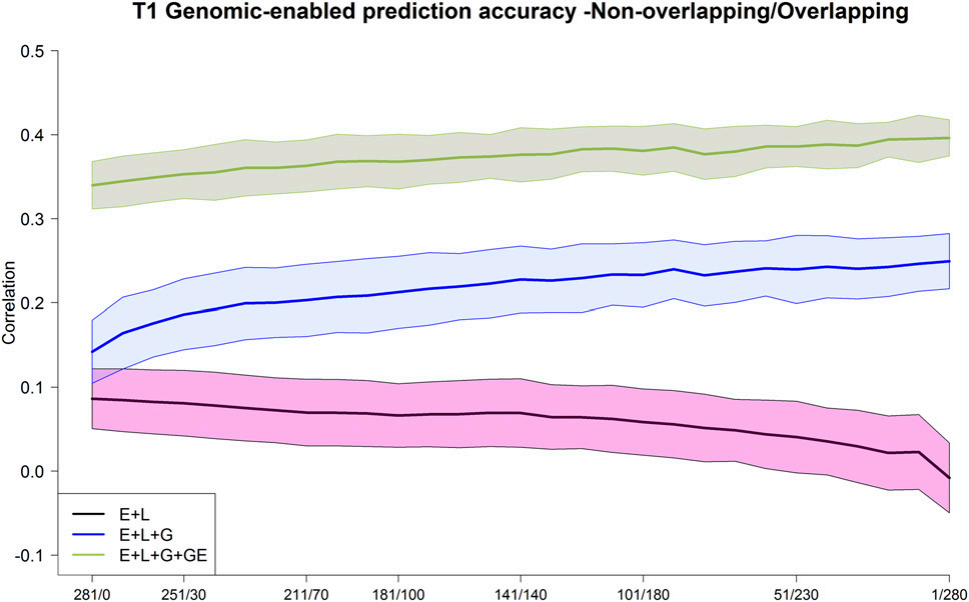
Maize data set DST1. Average Pearson’s correlation between the observed and predicted values of the maize genotypes for the 3 models M1 (E+L), M2 (E+L+G), and M3 (E+L+G+GE) for different composition of the non-overlapped/overlapped allocation designs. Shaded areas represent the interval of the mean plus minus one standard deviation.

**Figure 7:**
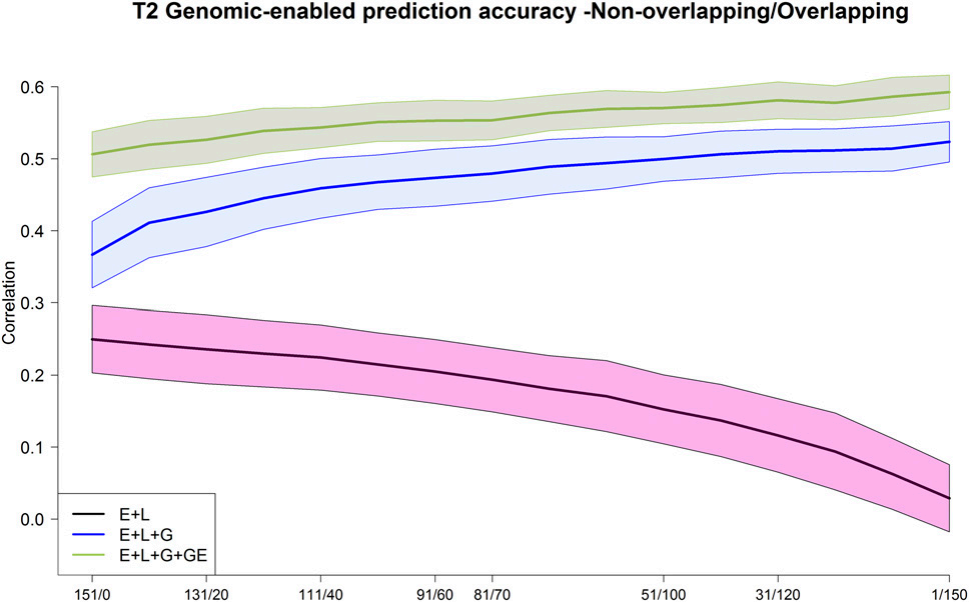
Maize data set DST2. Average Pearson’s correlation between the observed and predicted values of the maize genotypes for the 3 models M1 (E+L), M2 (E+L+G), and M3 (E+L+G+GE) for different composition of the non-overlapped/overlapped allocation designs. Shaded areas represent the interval of the mean plus minus one standard deviation.

**Figure A1:**
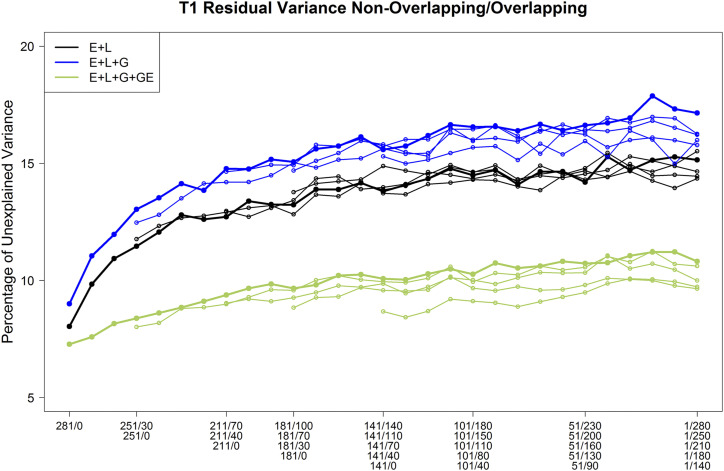
Maize data set DST1. Percent of the explained variance (residual variance) for the 3 models M1 (E+L), M2 (E+L+G), and M3 (E+L+G+GE) for different sizes and compositions of the non-overlapped/overlapped allocation designs. Thick lines represent the mean using the largest possible sample size for model calibration (281) for different compositions. The thinner lines represent the mean for the reduced/smaller sample sizes (281-141 in steps of 10).

Due to the extensive case for combining different repetitions (25), allocation sizes and composition of the NO/O allocation combinations, as well as different sizes of the initial populations, we present the mean of the results obtained with the largest allocation set (NO/O allocation compositions) for both data sets including all three prediction models in Figures 4-7. Detailed results for all of the different sizes are provided in Figures A1-A4 in the Appendix. Also, to make the presentation of the results clear and readable, we present the average of the mean accuracies across the three environments. The results regarding the percentage of the unexplained variance (residual variance) by the three models, and the corresponding interval of the mean plus or minus one standard deviation are presented as the mean of these components across the 25 repetitions for all cases.

### Percentage of unexplained variance (residual variance) resulting from the three prediction models

**Figure A2:**
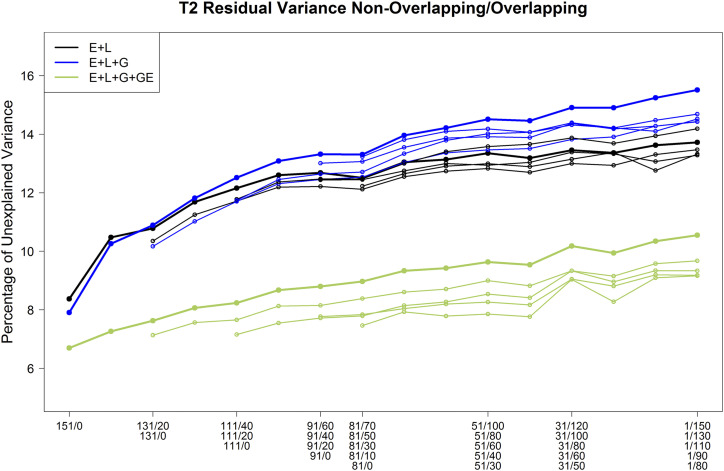
Maize data set DST2. Percent of the unexplained variance (residual variance) for the 3 models M1 (E+L), M2 (E+L+G), and M3 (E+L+G+GE) for different sizes and compositions of the non-overlapped/overlapped allocation designs. Thick lines represent the mean using the largest possible sample size for model calibration (151) for different compositions. The thinner lines represent the mean for the reduced/smaller sample sizes (151-81 in steps of 10).

The average percentage of the unexplained variability (residual variance) from M1-M3 are displayed in Figures 4 and A1 (DST1) and Figures 5 and A2 (DST2). The variance components were computed for each of the repetitions (25) and the combinations of the calibration set size - different NO/O allocation designs. For DST1 and DST2, the trends of the percentage of unexplained variance of the total variance showed differences as well as similarities across different allocation designs.

The patterns of the unexplained variance changed slightly with the testers. The residual variance from model **M1** was small (8∼9%) when each maize genotype was observed once across environments (left-hand side of the plots). Nevertheless, when the ratio of NO/O genotypes decreased (the number of common maize genotypes in the testing set was increased) (middle and right-hand sides of Figures 4-5 and A1-A2), the percentage of unexplained variability of **M1** consistently increased for both testers (14∼15%).

The associated residual variance from **M2** had a similar trend showing slightly larger values than **M1** in most cases. The residual variance of **M2** varied between 8% and 16% for both data sets (DST1 and DST2). Model **M3** returned the smaller percentage of residual variance in both data sets (DST1 and DST2). These values varied between 7% and 10% of the total variance.

As for the effect of the size of the allocation design (thick lines *vs.* thin lines of the same color in Figures A1-A2), in general, the residual variance of the **M3** model (thick green line *vs.* thin green lines) showed that green lines slightly increased the unexplained variance when more maize genotypes are common in all environments (lower ratio of NO/O genotypes when moving to the right-hand side of Figures A1-A2). Also, for model **M3**, the residual variance showed a smaller increase when reducing the sample size (green thin lines) compared with models **M1** and **M2**.

### Genome-based prediction accuracy of the various allocation designs

Figure 6 and Table 2 (for DST1), and Figure 7 and Table 3 (for DST2) show the average prediction accuracy across 25 replicates and all environments. Due to the large number of cases for training set size and set composition, in Table 2 we only use the headers of the largest data set; the information of the exact training composition can be found in Table 1.

**Table 2:** Maize tester T1. Average (across 3 environments) Pearson correlations (for 25 replicates) between the observed and predictive values for 3 models (M1-M3) for different sizes and composition of the non-overlapped/overlapped allocation design

	281/0	251/30	211/70	181/100	141/140	101/180	51/230	1/280
**M1 = E+L**								
281	0.086	0.081	0.070	0.066	0.069	0.058	0.041	−0.008
251		0.076	0.067	0.059	0.059	0.057	0.046	0.032
211			0.061	0.059	0.052	0.054	0.060	0.048
181				0.060	0.061	0.058	0.056	0.036
141					0.062	0.060	0.036	0.036
**M2 = E+L+G**								
281	0.142	0.186	0.203	0.213	0.228	0.233	0.240	0.249
251		0.139	0.189	0.201	0.215	0.227	0.233	0.239
211			0.139	0.184	0.200	0.208	0.221	0.226
181				0.141	0.188	0.200	0.211	0.217
141					0.152	0.193	0.193	0.206
**M3 = E+L+G+GE**								
281	0.340	0.353	0.363	0.368	0.376	0.381	0.386	0.396
251		0.329	0.345	0.353	0.361	0.368	0.370	0.379
211			0.321	0.335	0.346	0.351	0.367	0.366
181				0.312	0.329	0.338	0.352	0.353
141					0.304	0.313	0.322	0.338

**Table 3:** Maize tester T2. Average (3 environments) Pearson correlations (for 25 replicates) between the observed and predictive values for 3 models (M1-M3) for different sizes and composition of the non-overlapped/overlapped allocation design

	151/0	131/20	111/40	91/60	81/70	51/100	31/120	1/150
**M1 = E+L**								
151	0.250	0.236	0.224	0.205	0.181	0.152	0.116	0.029
131		0.217	0.203	0.193	0.173	0.150	0.129	0.099
111			0.155	0.141	0.125	0.104	0.095	0.100
91				0.089	0.096	0.105	0.106	0.091
81					0.101	0.104	0.101	0.075
**M2 = E+L+G**								
151	0.367	0.426	0.459	0.474	0.489	0.499	0.510	0.523
131		0.383	0.441	0.471	0.485	0.498	0.506	0.516
111			0.386	0.445	0.471	0.485	0.493	0.507
91				0.371	0.442	0.469	0.486	0.496
81					0.430	0.463	0.478	0.491
**M3 = E+L+G+GE**								
151	0.506	0.526	0.543	0.553	0.563	0.570	0.581	0.593
131		0.517	0.533	0.548	0.558	0.565	0.570	0.573
111			0.516	0.534	0.541	0.555	0.556	0.566
91				0.505	0.530	0.541	0.555	0.561
81					0.526	0.538	0.548	0.552

For DST1, results showed the clear superiority of model **M3** (green line) over models **M1** (black line) and **M2** (blue line) in terms of prediction accuracy. This superiority was accomplished for all combinations of allocation designs (NO/O). For models **M2** and **M3**, the prediction accuracy tends to increase as the ratio of NO/O becomes smaller. For example, for model **M3**, the genomic-enabled prediction accuracies at allocation combinations of 281/0, 141/140, and 1/280 were 0.340, 0.376 and 0.396, respectively (Table 2).

**Figure A3:**
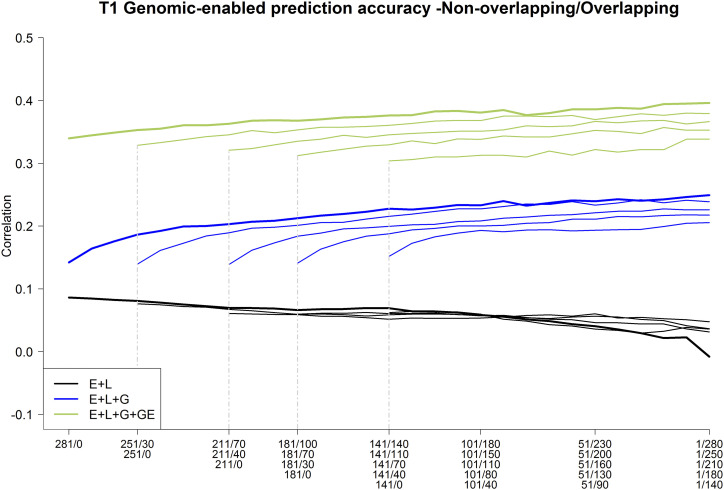
Maize data set DST1. Average Pearson’s correlation between the observed and predicted values of the maize genotypes for the 3 models M1 (E+L), M2 (E+L+G), and M3 (E+L+G+GE) for different sizes and compositions of the non-overlapped/overlapped allocation designs. Thick lines represent the mean using the largest possible sample size for model calibration (281) for different compositions. The thinner lines represent the mean for the reduced/smaller sample sizes (281-141 in steps of 10).

**Figure A4:**
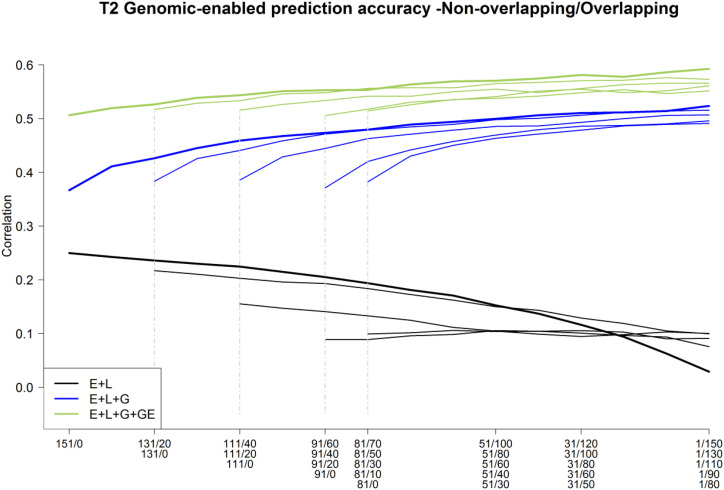
Maize data set DST2. Average Pearson’s correlation between the observed and predicted values of the maize genotypes for the 3 models M1 (E+L), M2 (E+L+G), and M3 (E+L+G+GE) for different sizes and compositions of the non-overlapped/overlapped allocation designs. Thick lines represent the mean using the largest possible sample size for model calibration (151) for different compositions. The thinner lines represent the mean for the reduced/smaller sample sizes (151-81 in steps of 10).

Regarding the sample size of the calibration sets (Figures A3-A4), when the number of genotypes evaluated in each environment decreased (251, 211, 181, 141), the genome-based prediction also decreased; however, a slight increasing trend was observed when more genotypes were commonly tested in all environments (reducing the NO/O ratio).

Similar trends in genome-based prediction accuracy were found for the data set involving maize tester T2. There is a clear superiority in terms of prediction accuracy of model **M3** (green line) over models **M1** (black line) and **M2** (blue line) (Figure 7) for the same combinations of allocation designs (NO/O). For model **M2** and **M3**, the genomic-enabled prediction accuracy increases as the ratio of NO/O genotypes decreases. For example, for model **M3**, the genomic-enabled prediction accuracies with allocation combinations 151/0, 81/70, and 1/150 were 0.506, 0.563, and 0.593, respectively (Table 3). When the sample size of the genotypes evaluated in each environment decreased (131, 111, 91, 81) (Figure 7 and Table 3), the genome-based prediction for models **M2**-**M4** remained practically unchanged with an increasing trend when reducing the NO/O ratio. This increase was more pronounced for **M2** than for **M3**; however, **M3** always delivered the best results.

In summary, for the two maize data sets, DST1 and DST2, the GE model **M3** was the best predictive model. These results were influenced by the size and composition of the allocation designs because there is a trend of increasing prediction accuracy as the number of common genotypes evaluated in environments increases, and there is a trend of decreasing accuracy when the sample size of the genotypes evaluated decreases, especially with DST1. The prediction accuracy for the DST2 data set for model **M3** reached almost 0.6 when all genotypes in the calibration set were tested in all three environments. For both data sets DST1 and DST2, prediction accuracies of models **M1** and **M2** were consistently lower than those achieved by model **M3**; however, also for model **M2**, the prediction accuracy increased when the NO/O ratio decreased.

These results suggest that it is better to have allocation designs consisting of a group of common genotypes repeated in all environments than to establish groups of different genotypes evaluated in all environments. Perhaps a common set of between 30 to 40 genotypes would provide acceptable improvements in predictive ability without the burden of seed availability. Obviously, this will depend, among other things, on the trait, the total number of genotypes and environments, and the availability of resources in general (phenotyping and genotyping costs).

## Discussion

Genomic-assisted breeding enables breeders to select genotypes in a better and more informed way with the main objective of increasing the expected genetic gain. This methodology has been described by many authors (Meuwissen *et al.* 2001; VanRaden 2008; de los Campos *et al.* 2009; Crossa *et al.* 2010). Some authors have explored the effects on predictive ability by varying the sample size of testing sets for a prediction set of fixed size for simulated (Lorenz 2013) and real data (Burgueño *et al.* 2012; Jarquín *et al.* 2014a). Other studies have considered the optimization of resources in multiple environments by blocking the GE interaction including mega-environments (González-Barrios *et al.* 2019) and spatial adjustments of phenotypes. However, in real applications, it is difficult to know the soil conditions in advance and successfully replicate the outcomes derived from these adjustments. To our knowledge, this is the first study that systematically assesses the effects on genomic-enabled predictive ability due to allocation designs where a certain number of different genotypes is distributed in different environments (non-overlapping) and another set of genotypes is repeatedly observed in all the environments (overlapping).

Results for both data sets measured for genomic prediction accuracy indicated that substantial savings could be achieved by overlapping a small number of genotypes in all environments (∼30 and 40) and allocating the rest of the genotypes in a non-overlapping design (NO = 251 and 111, respectively in these two data sets, Figures 6 and A3 and Figures 7 and A4) in different environments, especially when using the GE model (**M3**). This study showed that the prediction accuracy of GP increased or was stabilized when the ratio of the NO/O genotypes decreased. Clearly, the statistical model that included the GE component (**M3**) leveraged the information of genotypes tested in the target environments as well as in other environments. In this case, significant cost savings and increase in genome-based accuracy can be achieved by testing more common genotypes in all the environments with model **M3**. The **M3** model offers the advantage of returning accurate predictions for diverse calibration set compositions. The composition of the calibration sets depends on the seed availability for establishing trials in breeding programs, among other factors. Thus, we can state that **M3** easily adapts to the seed availability of the breeding programs when designing and planning field trials. This model also offers the advantage of increasing the capacity of evaluation of genotypes by delivering similar levels (moderate to high) of predictive ability with reduced sample sizes, allowing savings of resources (field, phenotyping cost, water use, etc.).

For example, for DST1, testing the same 280 maize genotypes in each of the 3 environments, and for DST2, testing the same 151 genotypes in the 3 environments produced higher prediction accuracy than other allocation methods using a GP model that includes the GE component (**M3**). However, researchers might like to include a small proportion of common genotypes across all environments to estimate the environmental variance (not to be confounded with the genotype variance) or, due to logistics, they might not have the desired materials to test in these environments but they might be evaluated in others.

In both maize data sets, the decrease in the size of the training set represented by thin lines (in the figures) had, as expected, a negative effect on the prediction accuracy, but when the ratio of NO/O genotypes decreased, the predictive ability of the models increased within the same training set sizes. These results can be explained by the smaller patterns of residual variance showed by **M3** for the DST1 and DST2 data sets.

### Predictive ability of the models used in this study

One objective of this research was to study different strategies for how to increase predictive ability by using allocation methods of genotypes with different proportions of NO/O in environments in conjunction with models that capture GE variance from the different sparse allocation testing designs. In model **M1**, for the disjointed partition (NO/O) (281/0 for DST1 and 151/0 for DST2), the effect of environments is confounded with the genotype effect; thus the prediction of an unobserved genotype in a particular environment is mainly influenced by the single observation (replicate) of that genotype but measured in a different environment. For model **M1**, the percentage of unexplained variance increased for low values of NO/O. Prediction accuracies followed opposite trends; as the residual variance of **M1** increased, when the NO/O proportion decreased, the predictive ability rapidly decreased.

The percentage of unexplained variance of model **M2** including genotypes and genomic information was the highest for both data sets for almost all of the cases (training set size and training composition), and the genomic-enabled prediction accuracy was intermediate between model **M1** and model **M3**. In general, DST2 gave higher prediction accuracy than DST1. The main reason why model **M3** was always the best predictive model resides in the fact that the GE interaction term reduced the unexplained proportion of the total variance significantly compared with the other models (**M1** and **M2**). Also, the GE term from model **M3** allows the borrowing of information from related genotypes evaluated in correlated environments.

It is possible to use other prediction models to leverage the GE interaction in prediction models for predicting unobserved genotypes. For example, the factor analytic model is a parsimonious model (Burgueño *et al.* 2012) for capturing the genetic correlations among environments. In addition, in this study, we used only genomic information, but it may be possible to add pedigree information, incorporated into **M3**; thus a slight increase in the prediction accuracy of the unobserved genotypes in the designs with different allocations can be still expected.

Furthermore, the type of marker system data (technology, platform, number of markers, cost, etc.) plays a relevant role in the assessment of sparse testing for genomic-enabled prediction. The high-density marker data used in this study (68,169 and 62,882 SNP markers for DST1 and DST2, respectively) were suitable for delivering appropriate genomic-enabled prediction accuracy. However, in a more realistic scenario, the cost of the markers must be also considered besides the total plot unit cost. Perhaps a high-density marker set becomes costly, and possibly a much lower marker density set would be necessary to stay within the boundaries of the budget. If the marker platform changes to low density, new studies will have to be conducted to consider their prediction accuracy as well as their total costs.

### Importance of sparse testing methods for genomic selection

As previously pointed out, there are different ways to study resource allocation in sparse testing. Obviously, plant breeding programs have limited financial resources per plot unit; thus, it is of paramount importance to plant only a limited number of plots while optimizing the molecular and field evaluation resources with the objective of increasing genetic gains. Therefore, given the fixed costs, breeders must study how many genotypes could be genotyped and how many of the total genotypes could be evaluated in the field, with the objective of designing allocation methods that save resources while increasing genetic gains. Some researchers aim to test more genotypes by using a sparse testing allocation method that focuses on increasing the intensity of selection, thus optimizing the response to selection. Other researchers aim for maximizing the genetic gains with a fixed plot unit cost but without increasing the intensity of selection, as enlarging field trials will inevitably increase the phenotyping costs.

Also, as pointed out, sparse testing schemes focusing on increasing the intensity of selection by increasing the number of testing genotypes will also increase the final genetic gains. Our study is directly related to increases in genetic gains because we show how the genetic and GE variance components change with different NO/O; however, our study did not directly assess increasing the intensity of selection as a factor for increasing genetic gains. Our study did not directly study the effect of an un-replicated (augmented) design in terms of costs influencing the NO/O ratio. However, some factors must be considered. One aspect of un-replicated designs is that they facilitate the increase in population size and thus the intensity of selection, but at the cost of diminishing the estimation precision. Another factor of un-replicated designs is the necessary balance between plots assigned to un-replicated entries *vs.* plots with replicated entries (or checks). Genomic-enabled prediction accuracy usually requires good and extensive phenotype data of the genotypes in the testing set.

## Conclusions

In this study, we evaluated the genomic-enabled prediction accuracy in different field sparse testing systems consisting of different ratios of NO/O genotypes included in environments. The results indicated that the genome-based model including GE captured more phenotypic variability (smaller residual variance) than the main effects models. In addition, the GE genomic model provided higher prediction accuracy than the main effects models in the different allocation designs comprising different combinations of NO/O genotypes in environments. Reducing the size of the testing populations slightly decreased the accuracy; however, the levels of predictive ability were recovered when we increased the number of common genotypes tested across environments. The GE model (**M3**) offers the possibility of maintaining the prediction accuracy when the two extreme situations occur [(1) all non-overlapping genotypes and (2) all overlapping genotypes)] while reducing the size of the training set. Results indicated that substantial savings of testing resources could be achieved by optimizing the allocation design using genome-based models including GE interaction. For the given sizes of the trials included in this study, it is recommended (but not necessary) to have a small proportion of genotypes overlapping in all the environments while a large proportion of genotypes should be non-overlapping in the environments.

## APPENDIX

Allocation designs varying the number of tested genotypes in different environments
